# Pleiotropism of the Photoperiod-Insensitive Allele of *Hd1* on Heading Date, Plant Height and Yield Traits in Rice

**DOI:** 10.1371/journal.pone.0052538

**Published:** 2012-12-20

**Authors:** Zhen-Hua Zhang, Kai Wang, Liang Guo, Yu-Jun Zhu, Ye-Yang Fan, Shi-Hua Cheng, Jie-Yun Zhuang

**Affiliations:** State Key Laboratory of Rice Biology and Chinese National Center for Rice Improvement, China National Rice Research Institute, Hangzhou, China; National Rice Research Center, United States of America

## Abstract

Five populations segregated in isogenic backgrounds and three sets of near isogenic lines (NILs) overlapping in a 362.3-kb region covering heading date gene *Hd1* were developed from the *indica* rice cross Zhenshan97 (ZS97)/Milyang 46 (MY46). They were used to analyze the effects of *Hd1* on heading date, plant height and yield traits. In a background of the parental mixtures, the photoperiod-sensitive allele derived from ZS97 functioned in promoting and delaying flowering in the natural short-day and long-day conditions, respectively. In the background of ZS97, no response to the photoperiod was observed, whereas the photoperiod-insensitive allele derived from MY46 functioned in delaying flowering, increasing plant height, and enhancing grain productivity. The additive effects estimated in two NIL sets were 6.14 and 6.14 d for heading date, 4.46 and 5.55 cm for plant height, 10.82 and 11.54 for the number of spikelets per panicle, 6.82 and 8.00 for the number of grains per panicle, and 2.16 and 2.23 g for grain yield per plant, which explained 94.1% and 96.3%, 70.5% and 84.8%, 52.4% and 55.2%, 28.9% and 39.2%, and 36.5% and 26.9% of the phenotypic variances, respectively. Since the photoperiod-insensitive allele of *Hd1* confers a long vegetative phase, it is a good candidate for breeding rice varieties with high yielding potential for low latitudes.

## Introduction

Heading date (HD) is the most crucial factor determining the regional and seasonal adaptation of rice (*Oryza sativa* L.). This trait is decided by basic vegetative phase (BVP), photoperiod sensitivity (PS) and temperature sensitivity (TS), among which PS plays the leading role as shown in molecular studies of quantitative trait loci (QTLs) underlying natural variation of flowering in rice. Of the eight QTLs that have been cloned, seven are involved in the photoperiodic control. *Hd3a* is the rice florigen for the PS pathway, with the expression being promoted in short-day (SD) conditions and suppressed in long-day (LD) conditions [1]. Six other QTLs involved in this pathway are *Hd1* and *Ehd1* that regulate *Hd3a* [2], [3], and *Hd6* [4], *Ghd7* [5], *DTH8*/*Ghd8* [6], [7] and *Hd17* [8] that regulate *Hd1* and/or *Ehd1*.

Nonetheless, the remaining QTL cloned for HD in rice, *DTH3*, appears to be not involved in the PS pathway, showing similar effects in SD and LD conditions [9]. Two of the QTLs involved in the PS pathway, *Hd1* and *Ehd1*, are also known to control the BVP. At the *Hd1* locus, the photoperiod-sensitive allele (PS allele) confers a short BVP and the non-sensitive allele (non-PS allele) confers a long BVP. At the *Ehd1* locus, the dominant allele *Ef1* confers a short BVP and the recessive alleles *ef1* and *ef-h* confer long BVP [10].

Increasing grain yield has been the most important objective of rice production. The duration of accumulating assimilation products are largely determined by heading date, while plant height (PH) is a key morphological trait related to yield potential. In populations that were used in QTL mapping for HD, PH and yield traits, it is not uncommon that QTLs for PH and/or yield traits were detected in regions where genes or QTLs for HD were located [11]. Among the eight QTLs cloned, *Ghd7* and *DTH8*/*Ghd8* were reported to have major effects on HD, PH and yield [5]–[7]. On the one hand, the association of enhancing grain yield with prolonging heading date might limit the regional and seasonal adaption of rice varieties [10]. On the other hand, long BVP is desirable for increasing the yield potential of rice in low-latitude regions and efforts have been made to identify genes responsible for long BVP [12], [13]. Investigation of the pleiotropism of genes controlling BVP in rice would help to establish an optimum breeding strategy for a specific ecological area.

In a previous study using populations derived from a residual heterozygote identified from a recombinant inbred population of the *indica* rice cross Zhenshan 97 (ZS97)/Milyang 46 (MY46), we found that a 1.90-Mb genomic region covering *Hd1* had significant effects on heading date and yield traits [14]. In the present study, five populations segregated in isogenic backgrounds and three sets of near isogenic lines (NILs) overlapping in a 362.3-kb region covering *Hd1* were developed and used to analyze the multiple effects of *Hd1* on heading date, plant height and yield traits.

## Materials and Methods

### Development of the Rice Materials

**Figure 1:**
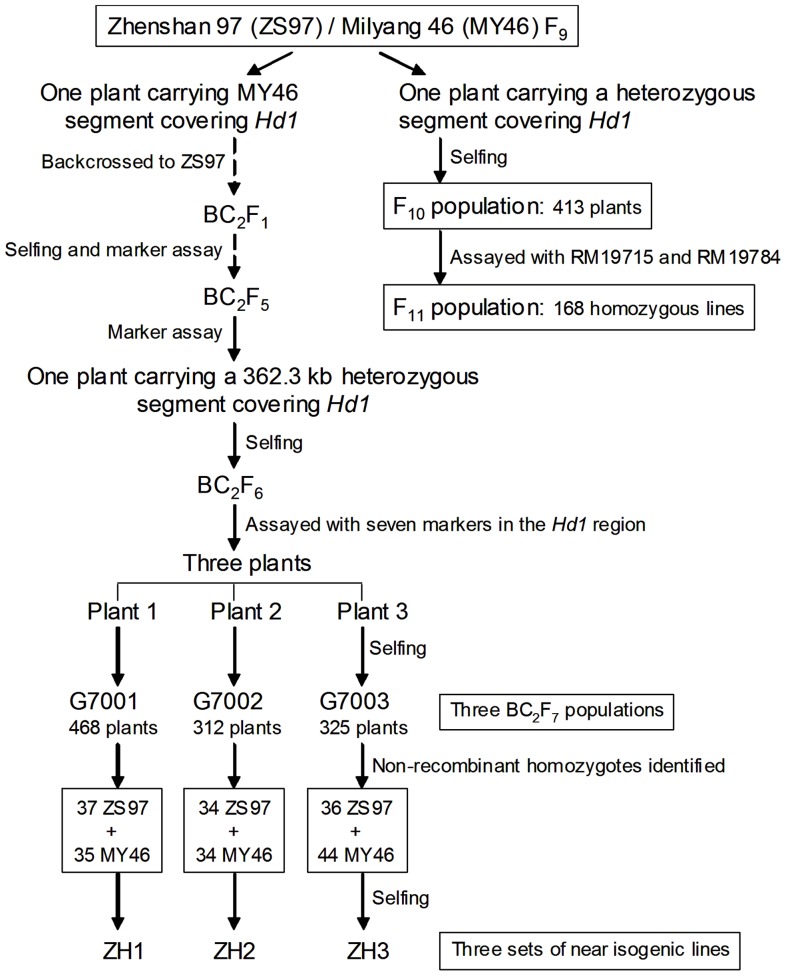
Development of the plant materials used in this study.

Five populations and three sets of near isogenic lines (NILs) were used in this study. Each of them was segregated in a region on the short arm of rice chromosome 6 that cover *Hd1* or is closely linked to *Hd1*. They were derived from the *indica* rice cross ZS97/MY46 as described below and summarized in Fig. 1. At the *Hd1* locus, ZS97 has the PS genotype of *Se-1^u^Se-1^u^* and MY46 has the non-PS genotype of *Se-1^e^Se-1^e^* [15].

An F_9_ plant of ZS97/MY46 carrying a heterozygous segment covering the *Hd1* region was selected. From selfed seeds of this plant, 413 F_10_ individual were produced and assayed using SSR markers RM19715 and RM19784 that flank the *Hd1*. Non-recombinant homozygotes were selected and an F_11_ population consisting of 168 lines was developed.

**Figure 2:**
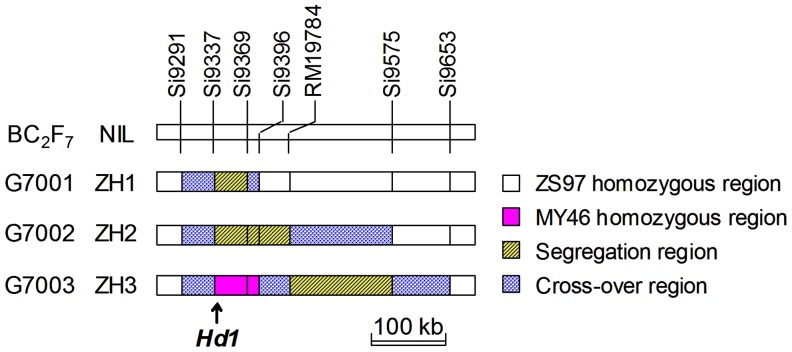
Genotypic composition of the BC_2_F_7_ populations and NILs in the target region.

Another F_9_ plant that carried a MY46 segment covering the *Hd1* region was selected and backcrossed to ZS97 for two times, followed by four generations of selfing. A BC_2_F_5_ plant carrying a 362.3-kb heterozygous segment covering the *Hd1* gene was identified. This plant was also found to be homozygous at 138 SSR marker loci that are polymorphic between the parental lines and located in background regions distributed on all the 12 chromosomes of rice. From selfed seeds of the BC_2_F_5_ plant, a BC_2_F_6_ population was derived and assayed with RM19784 and six new markers (Table 1) that are located in the 362.3-kb region. Three plants carrying overlapped heterozygous segments were identified, from which three BC_2_F_7_ populations were developed. They consisted of 468, 312 and 325 individuals and designated G7001, G7002 and G7003, respectively. While G7001 and G7002 segregated in regions Si9337-Si9369 and Si9337-RM19784 covering *Hd1*, respectively, G7003 was homozygous at the *Hd1* locus (Fig. 2).

**Table 1:** InDel and CAPS markers developed and used in this study.

Marker name	Marker type	Forward primer (5′-3′)	Reverse primer (5′-3′)	Restriction enzyme
Si9291	CAPS	CAAGGAAATTGACCATTAAGTTTGGCAC	GCTGGCGATCTTTCCACATACCG	*Bfa* I
Si9369	CAPS	CACGATGTTGACTTTTGGCAAT	GCTGCTTTCTAGTAATAGTCCTG	*Psh*B I
Si9396	CAPS	TGGATATCTCCTCTTTGGTGAATCGACGCT	CAAATTAAGCCGTAGTGCAT	*Tth*111 I
Si9337	InDel	AGATGTCCCTTCACTTCAGC	CGAAACGGCCCTTGATCC	–
Si9575	InDel	GCGCACACGGAGAACACC	ACAGCTCACGTATAAATGTGAACGA	–
Si9653	InDel	ACTGGATGTAACTATTGTATTGGCTA	GTCACACCGTCAGACCAT	–

The three BC_2_F_7_ populations were each assayed with two markers flanking their segregation regions, respectively, *i.e*., Si9337 and Si9369 for G7001, Si9337 and RM19784 for G7002, and RM19784 and Si9575 for G7003. Plants carrying non-recombinant homozygotes were identified and selfed to produce homozygous lines. Three sets of NILs were developed and named ZH1, ZH2 and ZH3 (Fig. 2). The numbers of ZS97 and MY46 homozygous lines included were 37 and 35 in ZH1, 34 and 34 in ZH2, and 36 and 44 in ZH3, respectively.

### Field Experiments

The rice populations were tested in the experiment stations of the China National Rice Research Institute located in either Lingshui of the Hainan Province or Hangzhou of the Zhejiang Province, China (Table 2). A planting density of 16.7 cm×26.7 cm was used for all the trials. During the floral transition period of the rice materials, the day length was shorter than 12.5 h in Lingshui and longer than 13.5 in Hangzhou (Figure S1) (Data was collected from www.timeanddate.com). Since the critical day length for triggering heading in rice is known to be 13.5 h [16], [17], day-length in Lingshui and Hangzhou are corresponding to natural SD and LD conditions, respectively.

**Table 2:** Field experiments conducted.

Generation	Name	Sample size	Location	Growing season	Trait measured^a^
F_10_	–	411 plants	Lingshui	Dec 2006–April 2007	HD
F_11_	–	168 lines	Hangzhou	May–Sep, 2007	HD
BC_2_F_7_	G7001	468 plants	Lingshui	Dec 2010–April 2011	HD
BC_2_F_7_	G7002	312 plants	Lingshui	Dec 2010–April 2011	HD
BC_2_F_7_	G7001	325 plants	Lingshui	Dec 2010–April 2011	HD
BC_2_F_8_	ZH1	72 lines	Hangzhou	May–Sep, 2011	HD, PH, yield traits
BC_2_F_8_	ZH2	68 lines	Hangzhou	May–Sep, 2011	HD, PH, yield traits
BC_2_F_8_	ZH3	80 lines	Hangzhou	May–Sep, 2011	HD, PH, yield traits

a HD, Heading date (d); PH, Plant height (cm); The yield traits measured are NSP (number of spikelets per panicle), NGP (number of grains per panicle), TGW (1000-grain weight, g), and GY (grain yield per plant, g).

The F_10_, F_11_ and three BC_2_F_7_ populations were measured for HD only, among which the F_10_ and three BC_2_F_7_ populations were scored on a single-plant basis, whereas the F_11_ lines were scored as the mean value over two replications. For the three NIL sets, a randomized complete block design was applied with two replications of eight plants per line. HD and PH were scored for each of the plants. At maturity, five middle plants of each line were harvested in bulk and measured for four yield traits (Table 2).

### DNA Marker Analysis

Total DNA was extracted following the method of Zheng et al [18]. PCR amplification was performed according to Chen et al [19]. The products of the two SSR and the three InDel markers were visualized on 6% non-denaturing polyacrylamide gels using silver staining, and that of the three CAPS markers were visualized on 2% agrose gels using Gelred staining. All of the SSR markers were selected from the Gramene database (www.gramene.org). The CAPS and InDel markers were designed using Oligo Primer Analysis Software Version 7.0 [20] based on SNPs and InDels between ZS97 and MY46 detected by the whole-genome re-sequencing. While Si9337 detected a 4-bp InDel in the exon 2 of the *Hd1* gene (Figure S2), other markers were located outside the *Hd1* locus.

### Data Analysis

Linkage map construction and QTL analysis were performed for the F_10_, F_11_ and BC_2_F_7_ populations. The maps were constructed using Mapmaker/Exp 3.0 [21]. Distances between markers were presented in centiMorgan (cM) derived using Kosambi function. QTLs were determined with the composite interval mapping of Windows QTL Cartographer 2.5 [22].

Two-way ANOVA was conducted to test the phenotypic differences between the two genotypic groups in each of the three NIL sets. The analysis was performed with SAS procedure GLM [23] as described previously [24]. Given the detection of significant difference (*P*<0.01), the same modal was applied to estimate the additive effect and the proportion of phenotypic variance explained.

## Results

### The Effect of *Hd1* on Heading Date

The F_10_ plants grown in Lingshui displayed a discontinuous distribution of 312 early-heading (87–91 d) and 99 late-heading (97–104 d), fitting the 3∶1 ratio for a single dominance gene (*P* = 0.67). The major effect of the *Hd1* region on heading date was confirmed by QTL analysis using the segmental linkage map spanning 5.8 cM. It accounted for 98.2% of the phenotypic variance, with the ZS97 allele promoting heading date by 6.99 d (Table 3).

**Table 3:** QTL analysis for heading date in the F_10∶11_ population and three BC_2_F_7_ populations.

Generation	Name	Location	Segregation region	Phenotypic mean(mean±SD)			LOD	A^a^	D^b^	R^2^(%)^c^
				ZS97	MY46	Heterozygote				
F_10_		Lingshui	RM19715–RM19784	88.83±0.63	102.62±1.78	89.19±0.98	370.54	6.99	−6.70	98.2
F_11_		Hangzhou	RM19715–RM19784	103.77±0.88	89.96±1.29	–	149.88	−6.98	–	97.6
BC_2_F_7_	G7001	Lingshui	Si9337–Si9396	94.90±2.26	103.94±3.36	96.14±2.37	106.46	4.64	−3.46	67.8
BC_2_F_7_	G7002	Lingshui	Si9337–RM19784	94.87±2.57	105.55±3.41	96.65±2.83	68.38	5.34	−3.54	63.4
BC_2_F_7_	G7003	Lingshui	RM19784–Si9575	105.44±2.60	105.50±2.54	105.56±2.33	0.03			

a Additive effect of replacing a Zhenshan 97 (ZS97) allele by a Milyang 46 (MY46) allele.

b Dominance effect.

c Proportion of phenotypic variance explained by the QTL effect.

The major effect was also detected in the F_11_ population grown in Hangzhou, but the allele for promoting heading date was derived from MY46. The effect explained 97.6% of the phenotypic variance, with the ZS97 allele delayed heading date by 6.98 d (Table 3).

In the two BC_2_F_7_ populations that segregated the *Hd1* locus and grown in Lingshui, G7001 and G7002, continuous distribution for heading date was observed although the ZS97 homozygotes tend to flower earlier than MY46 homozygotes. The additive effect of *Hd1* and its contribution to the phenotypic variance were estimated to be 4.64 d and 67.8% in G7001, and 5.34 d and 63.4% in G7002, respectively (Table 3), which are much lower than the effects detected in the F_10_ and F_11_ populations. As expected, no significant effect on heading date was detected in G7003 that was segregated in a region excluding the *Hd1* locus.

Among the three NIL sets grown in Hangzhou, significant variations on heading date were detected in ZH1 and ZH2 that were heterogenous at the *Hd1* locus. The additive effect of *Hd1* and its contribution to the phenotypic variance were estimated to be 6.14 d and 94.1% in ZH1, and 6.14 d and 96.3% in ZH2, respectively (Table 3). As expected, no significant effect on heading date was detected in ZH3 that was homogenous at the *Hd1* locus.

Worthy of note, the allele for promoting heading date were all derived from ZS97 in the two BC_2_C_7_ populations and two NIL sets, although the BC_2_C_7_ populations were grown in Lingshui and the NIL sets in Hangzhou. This is obviously different from the results obtained from the F_10_ and F_11_ populations.

### The Effect of *Hd1* on Plant Height and Yield Traits

As described above, the effect of the *Hd1* was detected in the NIL sets ZH1 and ZH2. As shown in Fig. 2, the common heterogenous region of the two NIL sets extended from markers Si9337 to Si9369. As referred to the physical positions in the Nipponbare genome (www.gramene.org), Si9337 is located at 9,337,119–9,337,269 bp and Si9369 at 9,369,434–9,369,808 bp of the rice chromosome 6. In addition to *Hd1* (*LOC_Os06g16370*), four annotated genes, *LOC_Os06g16380*, *LOC_Os06g16390*, *LOC_Os06g16400*, and *LOC_Os06g16410*, were located in this region. None of the four annotated genes have been shown to affect heading date, plant height and yield traits in rice. Additionally, no gene for plant height and yield traits has been cloned or fine-mapped in this region, thus the effects on plant height and yield traits detected in the two NIL sets would provide an evidence for the pleiotropism of the *Hd1* gene.

Result of the two-way ANOVA on the phenotypic differences between the two genotypic groups in each of the three NIL sets were presented in Table 4. Significant variations were found in the NIL sets ZH1 and ZH2 for all the traits analyzed except for TGW in ZH1. Both the frequency distribution (Figure S3) and the results of ANOVA indicated that the *Hd1* region had major effects on PH, NSP, NGP and GY. Moreover, phenotypic variations and the genetic effects detected for each of the traits were similar over the two NIL sets. In ZH1 and ZH2, the contribution to the phenotypic variance were 70.5% and 84.8% for PH, 52.4% and 55.2% for NSP, 28.9% and 39.2% for NGP, and 36.5% and 26.9% for GY, with the MY46 allele increasing PH by 4.46 and 5.55 cm, NSP by 10.82 and 11.54, NGP by 6.82 and 8.00, and GY by 2.16 and 2.23 g, respectively. For TGW, significant effect was only detected in ZH2, with MY46 allele increasing TGW by 0.30 g.

**Table 4:** Analysis of variance for heading date, plant height and five yield traits in three sets of near isogenic lines (NILs).

NIL set	Segregation region	Trait	Phenotypic mean(mean±SD)		*P*	A	R^2^(%)
			ZS97	MY46			
ZH1	Si9337-Si9396	HD	63.76±0.97	76.03±1.22	<0.0001	6.14	94.1
		PH	94.03±1.50	103.16±1.87	<0.0001	4.46	70.5
		NSP	120.34±6.29	142.07±8.07	<0.0001	10.82	52.4
		NGP	101.16±5.88	114.87±9.06	<0.0001	6.82	28.9
		TGW	27.06±0.28	27.04±0.53	0.9067		
		GY	23.06±1.38	27.39±2.77	<0.0001	2.16	36.5
ZH2	Si9337-RM19784	HD	65.69±1.06	77.96±0.76	<0.0001	6.14	96.3
		PH	92.95±1.59	104.05±2.19	<0.0001	5.55	84.8
		NSP	119.48±6.13	142.56±8.51	<0.0001	11.54	55.2
		NGP	99.46±6.71	115.46±7.26	<0.0001	8.00	39.2
		TGW	25.98±0.35	26.58±0.53	<0.0001	0.30	20.3
		GY	22.40±2.59	26.85±3.33	<0.0001	2.23	26.9
ZH3	RM19784-Si9575	HD	78.38±0.93	78.39±0.88	0.9478		
		PH	105.61±1.69	105.51±1.52	0.7802		
		NSP	151.36±7.90	152.44±6.43	0.5081		
		NGP	116.70±8.23	117.85±8.35	0.5484		
		TGW	26.32±0.34	26.45±0.55	0.2192		
		GY	22.04±2.11	22.09±2.65	0.9090		
Parental lines		HD	67.50±0.94	80.67±0.71			
		PH	92.70±0.42	94.20±3.96			
		NSP	129.97±3.91	125.82±12.79			
		NGP	102.83±0.66	95.97±13.34			
		TGW	27.08±0.12	24.60±0.07			
		GY	25.76±3.05	22.85±9.50			

## Discussion

Although many regions covering QTLs for HD exhibits significant effects on PH and yield traits in primary mapping, the pleiotropism has only been confirmed for *Ghd7* and *DTH8*/*Ghd8.* In the present study, we demonstrated that the key HD gene *Hd1* has major effects on HD, PH and yield traits in the genetic background of the *indica* rice variety ZS97. Similar to *Ghd7* and *DTH8*/*Ghd8* [5]–[7], *Hd1* affects grain yield primarily because of its influence on NSP and NGP. Nevertheless, our study showed that *Hd1* might also affect TGW although the effects are smaller and less consistent.

It has been shown that *Hd1* exhibits dual functions on the flowering of rice depending on the day length, which promotes heading under SD conditions but is converted to represses heading in LD conditions [25]. In the F_10_ and F_11_ populations of ZS97/MY46, the ZS97 allele at *Hd1* promoted heading in natural SD condition (Lingshui) and delayed heading in natural LD condition (Hangzhou), which is in accordance with the understanding that ZS97 and MY46 carries PS and non-PS alleles at the *Hd1* locus, respectively [15].

Breeding rice varieties with long BVP and weak PS has been consider an important strategy in low latitudes where short photoperiods persist throughout the year. In the BC_2_F_7_ and BC_2_F_8_ populations of ZS97/MY46, the non-PS allele at *Hd1* which was derived from MY46 delayed heading in both the natural SD and LD conditions, which is in accordance with the understanding that non-PS allele of the *Hd1* confers a long BVP [10]. Moreover, the non-PS allele from MY46 was found to be associated with increases in grain number, providing a good candidate for breeding rice varieties with high yielding potential for low latitudes.

It is noteworthy that the sensitivity to photoperiod of the ZS97 allele of *Hd1* observed in the F_10∶11_ population disappeared in the BC_2_F_7∶8_ populations. It was found that the F_10∶11_ individuals are similar to MY46 with 73.1% of 207 polymorphic markers in the background showing MY46 genotype, while the BC_2_F_7∶8_ individuals are largely identical to ZS97 with 95.7% of 138 polymorphic markers in the background showing ZS97 genotype (data not shown). This suggests that the effect of the *Hd1* is not only determined by the environmental conditions but also depends on the genetic background. The similar phenomenon was observed in *se5* mutant, in which the null allele of *Hd1* consistently promoted heading in both SD and LD conditions [26]. Such a phenomenon was also observed in *phyB* mutant, in which delaying HD caused by over-expression of the *Hd1* under SD conditions was eliminated [17].

It has been reported that the *Hd1* gene functions in the evolutionarily conserved *OsGI*-*Hd1*-*Hd3a* pathway for the photoperiodic control of flowering in rice [27]. Based on the genotypes of DNA markers used for background detection in our studies, it was found that the F_10∶11_ populations carried MY46 homozygotes in the region covering *OsGI* on the short arm of rice chromosome 1, whereas the BC_2_F_7∶8_ populations carried ZS97 homozygotes in this region. Since ZS97 is an early season rice cultivars which is insensitive to photoperiod, our results suggest that ZS97 carries a non-functional allele at *OsGI*, thus the PS-allele carried by ZS97 does not response to photoperiod in the BC_2_F_7∶8_ populations.

## Supporting Information

Figure S1 **Day-length in Lingshui and Hangzhou during the period from sowing to last heading.**
(TIF)

Figure S2 **Sequence of the ZS97 allele in exon 2 of the** ***Hd1*** **gene.** Positions of the Si9377 primers are indicated by green characters, and the four nucleotide deleted in MY46 are indicated by yellow characters.
(TIF)

Figure S3 **Distribution of six traits in the NIL sets ZH1 and ZH2.**
(TIF)
