# Supplementary figures and images for: Pleiotropism of the Photoperiod-Insensitive Allele of Hd1 on Heading Date, Plant Height and Yield Traits in Rice
Source: PLoS One. 2012 Dec 20;7(12):e52538. doi: 10.1371/journal.pone.0052538 (PMC3527549; doi:10.1371/journal.pone.0052538)

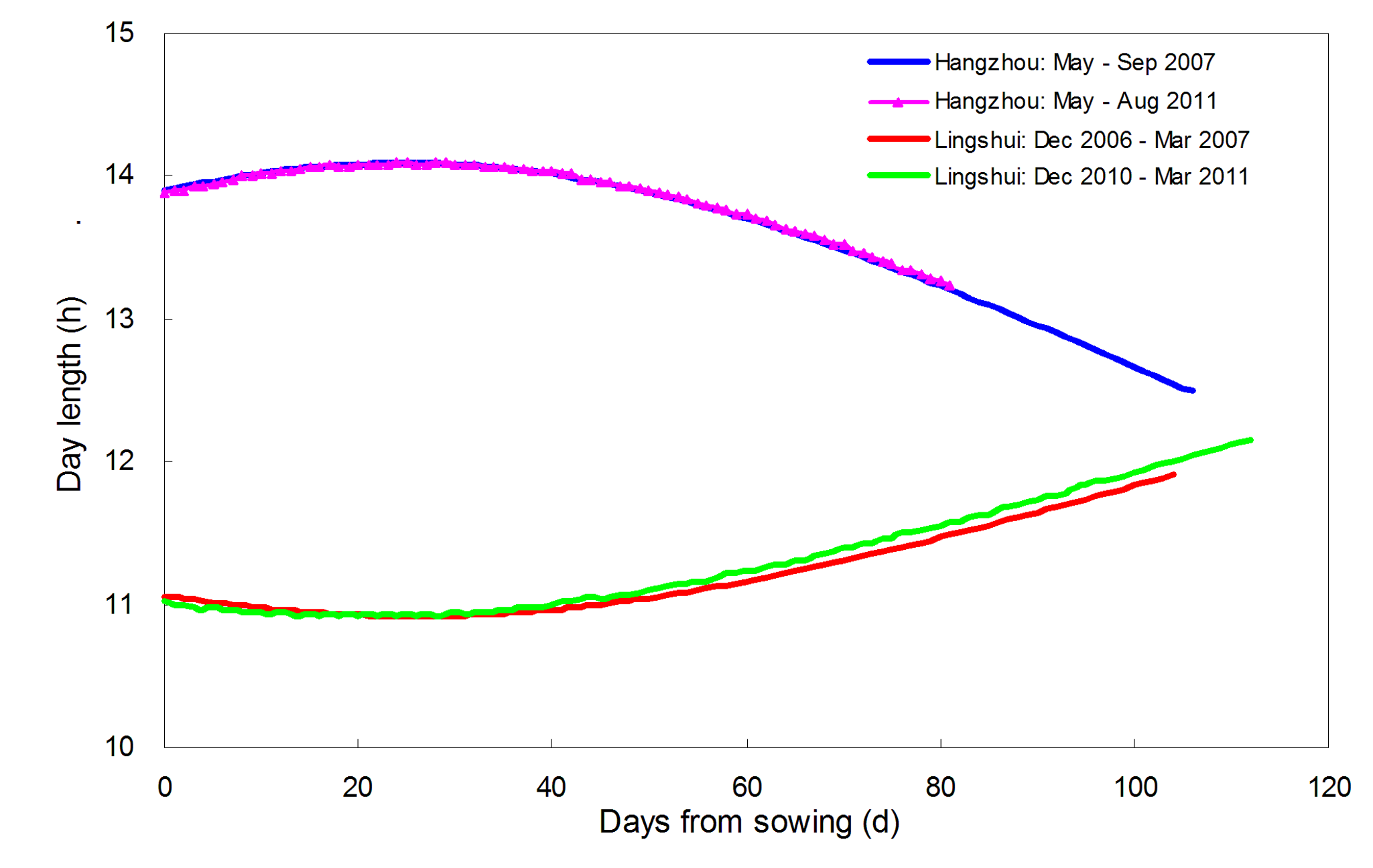

Supplement: Figure S1 — Day-length in Lingshui and Hangzhou during the period from sowing to last heading. (TIF) [file pone.0052538.s001.tif]

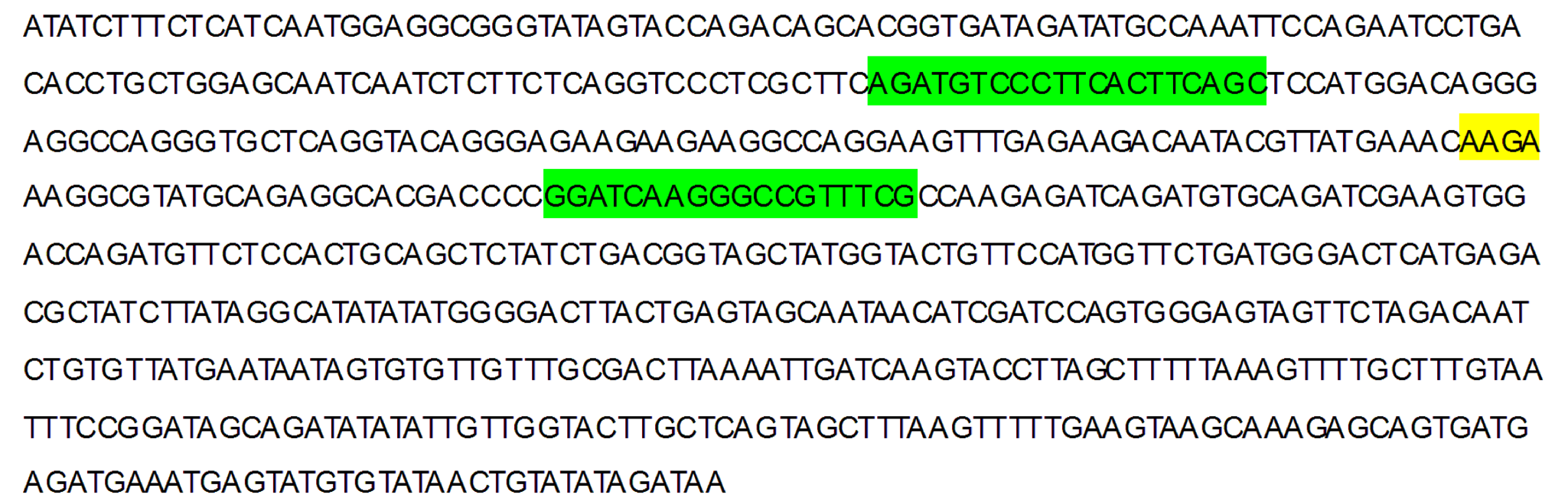

Supplement: Figure S2 — Sequence of the ZS97 allele in exon 2 of the Hd1 gene. Positions of the Si9377 primers are indicated by green characters, and the four nucleotide deleted in MY46 are indicated by yellow characters. (TIF) [file pone.0052538.s002.tif]

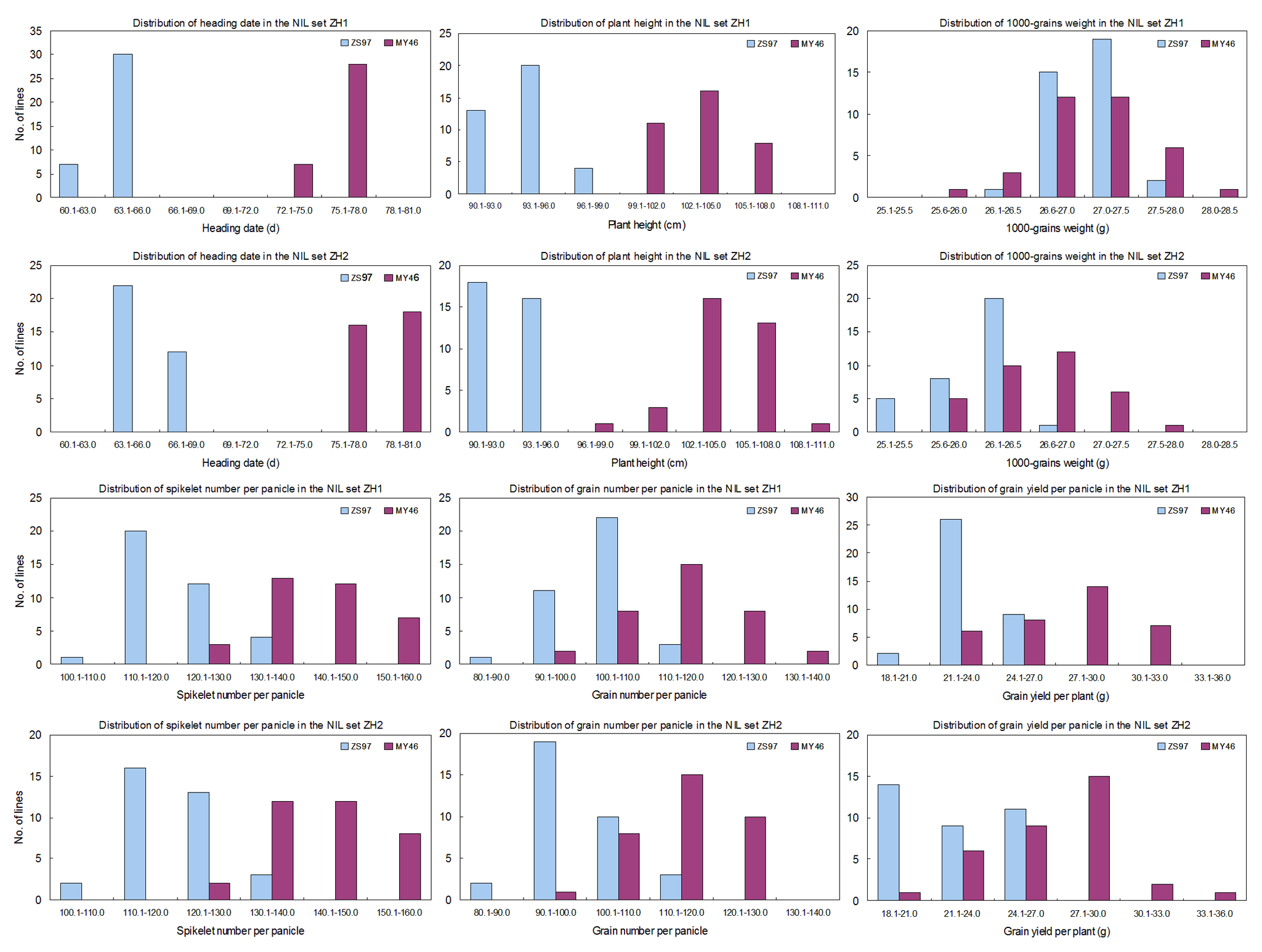

Supplement: Figure S3 — Distribution of six traits in the NIL sets ZH1 and ZH2. (TIF) [file pone.0052538.s003.tif]
